# Racial/Ethnic Disparities in the Prevalence of Selected Chronic Diseases Among US Air Force Members, 2008

**DOI:** 10.5888/pcd9.110136

**Published:** 2012-06-14

**Authors:** Jennifer J. Hatzfeld, Thomas A. LaVeist, Fannie G. Gaston-Johansson

**Affiliations:** Author Affiliations: Thomas A. LaVeist, Johns Hopkins Bloomberg School of Public Health, Baltimore, Maryland; Fannie G. Gaston-Johansson, PhD, RN, FAAN, Johns Hopkins School of Nursing, Baltimore, Maryland.

## Abstract

**Introduction:**

Few studies have evaluated possible racial/ethnic disparities in chronic disease prevalence among US Air Force active-duty members. Because members have equal access to free health care and preventive screening, the presence of health disparities in this population could offer new insight into the source of these disparities. Our objective was to identify whether the prevalence of 4 common chronic diseases differed by race/ethnicity in this population.

**Methods:**

We compiled de-identified clinical and administrative data for Air Force members aged 21 or older who had been on active duty for at least 12 months as of October 2008 (N = 284,850). Multivariate logistic regression models were used to determine the prevalence of hypertension, dyslipidemia, type 2 diabetes, and asthma by race/ethnicity, controlling for rank and sex.

**Results:**

Hypertension was the most prevalent chronic condition (5.3%), followed by dyslipidemia (4.6%), asthma (0.9%), and diabetes (0.3%). Significant differences were noted by race/ethnicity for all conditions. Compared with non-Hispanic whites, the prevalence of all chronic diseases was higher for non-Hispanic blacks; disparities for adults of other minority race/ethnicity categories were evident but less consistent.

**Conclusion:**

The existence of racial/ethnic disparities among active-duty Air Force members, despite equal access to free health care, indicates that premilitary health risks continue after enlistment. Racial and ethnic disparities in the prevalence of these chronic diseases suggest the need to ensure preventive health care practices and community outreach efforts are effective for racial/ethnic minorities, particularly non-Hispanic blacks.

## Introduction

Non-Hispanic black active-duty members of the military are 87% more likely to be hospitalized for asthma (1) and twice as likely to develop type 2 diabetes (2) as their non-Hispanic white counterparts. Similarly, compared with non-Hispanic whites, Hispanics are 29% more likely to be hospitalized for asthma (1) and 60% more likely to develop diabetes (2). Outside of the military, racial/ethnic disparities have been consistently identified in the US health care system (3), particularly in the prevalence of hypertension (4,5), dyslipidemia (4,6), diabetes (7,8), and asthma (9,10).

Throughout the US Air Force (USAF) health care system, standards for health care access are the same, regardless of geographic area, which helps to ensure each active-duty member receives the same level of health care. Additionally, every USAF active-duty member has at least a high school diploma and has been screened for pre-existing health conditions; once on active duty, each member is provided an equal housing benefit (based on rank) and has the same community resources, regardless of race/ethnicity. Collectively, the active-duty population in the USAF provides a unique opportunity to examine the presence of health disparities in a homogenous population of different racial/ethnic backgrounds.

Despite some evidence of disparities in asthma control and diabetes incidence, each military member receives the same comprehensive health care benefits with a regular household income, adequate housing, and an additional food allowance as part of his or her military benefits. Because of these factors, we hypothesized that no clinically significant disparities would be noted.

Although consistent racial/ethnic disparities in chronic diseases have been identified outside of the military, and 2 studies have found disparities for military members in asthma hospitalization and the incidence of type 2 diabetes, the prevalence of asthma and diabetes, by race/ethnicity, has not been established. Similarly, disparities in prevalence of cardiovascular diseases, including hypertension and dyslipidemia, have not been determined in the military population. The purpose of this study was to determine whether disparities exist in the prevalence of hypertension, dylipidemia, diabetes, and asthma among active-duty USAF members.

## Methods

We conducted a secondary analysis of existing clinical and administrative data for this descriptive, correlational study. The institutional review board (IRB) at Johns Hopkins University, the USAF Surgeon General’s Research Oversight and Compliance Division, the IRB at the Uniformed Services University of the Health Sciences, and the USAF Clinical Informatics Branch approved the study. The USAF Clinical Informatics Branch compiled data in October 2008 for the preceding 24 months.

### Chronic disease screening

Active-duty members are carefully screened before enlistment to ensure they are healthy enough for a military deployment (11). Annually, each active-duty member completes a preventive health assessment, which evaluates any new diagnoses received during the previous year and screens for chronic diseases, as recommended by the US Preventive Services Task Force. Any new medical diagnoses are also noted at that time and reviewed by the provider to determine the need for a Medical Evaluation Board, a process designed to determine whether soldiers’ long-term medical conditions enable them to continue to meet medical retention standards. The Medical Evaluation Board also provides the opportunity for military physicians to clearly document soldiers’ medical conditions and any duty limitations these may cause. All clinical information is maintained in a Department of Defense electronic medical record system and tracked in the Air Force medical readiness database (11).

### Study variables

We compiled de-identified clinical and administrative data from all USAF members aged 21 or older and on active duty at least 12 months as of October 2008 (N = 284,850). Higher ranks (1-, 2-, 3-, and 4-star generals) were not included in the original sample because of their small numbers (n = 204) and the personalized health care support they receive. Members on active duty less than 12 months were also excluded because newly enlisted or commissioned members have up to 12 months to get an annual preventive health assessment (11). Members with a rank of “other” (n = 1,120) and/or a race/ethnicity category of “other/declined” (n = 6,762) were subsequently excluded from further analysis, for a final sample size of 277,001.

Sex, race/ethnicity, and rank category were identified through the Defense Enrollment Eligibility Reporting System. Five categories were used to identify race/ethnicity: American Indian/Alaska Native, Asian/Pacific Islander, non-Hispanic black, non-Hispanic white, and Hispanic. Rank was also categorized to ensure that people in the data set could not be identified by their demographic information. The 4 rank categories were junior enlisted (from airman basic [E-1] through senior airman [E-4]), senior enlisted (from staff sergeant [E-5] through chief master sergeant [E-9]), junior officer (from second lieutenant [O-1] through captain [O-3]), and senior officer (from major [O-4] through colonel [O-6]). A category of Other included remaining enlisted personnel (eg, special agents).

Hypertension was identified for members with 2 or more medical appointments with a credentialed provider in the previous 24 months resulting in a primary diagnosis assigned an *International Classification of Diseases, Ninth Revision, Clinical Modification* (ICD-9-CM) code beginning with 401 (12). The clinical definition for the diagnosis was based on the Seventh Report of the Joint National Committee on Prevention, Detection, Evaluation, and Treatment of High Blood Pressure as a blood pressure reading above 140/90 mm Hg (13). Likewise, dyslipidemia was identified for members with 2 or more medical appointments during the previous 24 months with a primary diagnosis of dyslipidemia (ICD-9-CM code 272.4) (12), using the clinical definition from the National Heart, Lung, and Blood Institute Detection, Evaluation, and Treatment of High Blood Cholesterol in Adults guidelines (14). Members were identified as having diabetes or asthma using the National Committee for Quality Assurance’s Health Plan Employer Data and Information Set criteria (15). According to these criteria, diabetes is defined by 2 outpatient encounters with a diagnosis of diabetes (ICD-9-CM code beginning with 250), 1 emergency department or inpatient admission with diabetes, or receiving insulin or oral hypoglycemic/antihyperglycemic medications — excluding patients with a diagnosis of gestational diabetes, polycystic ovaries, steroid-induced diabetes, or prediabetes (15). Similarly, asthma was identified for members who had a least 4 medication-dispensing events, 4 outpatient visits for asthma (ICD-9-CM code beginning with 493) with at least 2 medication-dispensing events, or 1 emergency department or inpatient admission with a principal diagnosis of asthma (15).

Appointment data, including medical diagnosis, are entered into the Standard Ambulatory Data Record according to individual facility processes and are audited monthly to ensure accuracy. This audit is centrally directed, and health care leaders at all levels in the USAF Medical Service oversee the coding accuracy results. Together with the careful monitoring of health care quality metrics, these efforts have maintained a high quality of clinical and administrative data (16).

### Statistical analysis

We summarized demographic characteristics and prevalence of the 4 chronic diseases using descriptive statistics. We then created multivariate logistic regression models to calculate the prevalence of each chronic disease, adjusting for rank and sex.

Age had a strong nonlinear relationship with the prevalence of chronic diseases; therefore, we created spline terms at each 5-year interval, through age 40. This age effect differed by race/ethnicity, sex, and rank, so interaction terms for age and each of these independent variables were tested for significance. After a final model was determined for each outcome, the predicted prevalence for each race/ethnicity, by age range, was calculated with 95% confidence intervals.

## Results

More than half of the study population was aged 30 or younger, and the overall prevalence of the chronic diseases studied was low; hypertension was the most prevalent (5.3%), followed by dyslipidemia (4.6%), asthma (0.9%), and diabetes (0.3%) (Table 1). After adjustment for sex and rank, the prevalence of all 4 conditions differed significantly by race/ethnicity and was positively associated with age; racial/ethnic differences in prevalence also increased with increasing age (Table 2).

**Table 1:** Demographic Characteristics and Prevalence of Chronic Diseases Among All Active-Duty US Air Force Members (N = 284,850),^a^ 2008

Characteristic	n (%)
**Race/ethnicity**	
American Indian/Alaska Native	2,544 (0.9)
Asian/Pacific Islander	13,295 (4.7)
Black, non-Hispanic	41,970 (14.7)
White, non-Hispanic	203,857 (71.6)
Hispanic	16,422 (5.8)
Other/declined to respond	6,762 (2.4)
**Sex**	
Male	230,579 (80.9)
Female	54,271 (19.0)
**Age, y**	
21-25	85,881 (30.1)
26-30	70,318 (24.7)
31-35	47,999 (16.9)
36-40	43,519 (15.3)
≥41	37,133 (13.0)
**Rank category**	
Junior enlisted (E1-E4)	89,377 (31.4)
Senior enlisted (E5-E9)	132,821 (46.6)
Junior officer (O1-O3)	35,500 (12.5)
Senior officer (O4-O6)	26,032 (9.1)
Other (eg, special agent)	1,120 (0.4)
**Chronic diseases^b^**	
Hypertension	15,128 (5.3)
Dyslipidemia	12,987 (4.6)
Diabetes	892 (0.3)
Asthma	2,477 (0.9)

^a^ Aged ≥21 and on active duty ≥12 months (excluding officers with the rank of general).

^b^ Defined by the presence of appropriate *International Classification of Diseases, Ninth Revision, Clinical Modification* codes in the medical record for the preceding 2 years (12).

**Table 2:** Prevalence of Chronic Diseases, by Race/Ethnicity, Among Active-Duty US Air Force Members, 2008^a^

Chronic Disease^b^/Age	Non-Hispanic White, % (95% CI) (n = 203,015)	American Indian/Alaska Native, % (95% CI) (n = 2,533)	Asian/Pacific Islander, % (95% CI) (n = 13,253)	Non-Hispanic Black, % (95% CI) (n = 41,861)	Hispanic, % (95% CI) (n = 16,339)
**Hypertension**					
21-25 y	0.98 (0.98-0.99)	1.22 (1.19-1.25)	0.86 (0.86-0.87)	1.87 (1.86-1.89)	0.81 (0.80-0.83)
26-30 y	2.24 (2.23-2.25)	2.67 (2.59-2.75)	2.03 (2.00-2.07)	4.84 (4.81-4.88)	1.98 (1.96-1.99)
31-35 y	4.22 (4.21-4.24)	5.42 (5.23-5.62)	5.51 (5.41- 5.61)	9.53 (9.46-9.61)	4.17 (4.12-4.22)
36-40 y	8.15 (8.12-8.19)	10.02 (9.61-10.42)	10.96 (10.74-11.18)	17.61 (17.48-17.75)	8.14 (8.03-8.26)
≥41 y	12.92 (12.87-12.98)	14.75 (14.23-15.27)	18.3 (17.93-18.66)	27.6 (27.41-27.79)	14.31 (14.06-14.57)
Overall	4.57 (4.55-4.59)	4.5 (4.32-4.68)	4.62 (4.52-4.72)	9.57 (9.48-9.66)	4.33 (4.26-4.41)
**Dyslipidemia**					
21-25 y	0.55 (0.55-0.55)	0.76 (0.74-0.79)	0.80 (0.79-0.81)	0.65 (0.64-0.65)	0.76 (0.75-0.77)
26-30 y	1.64 (1.64-1.65)	2.2 (2.13-2.27)	2.24 (2.21-2.27)	1.82 (1.80-1.83)	1.98 (1.95-2.00)
31-35 y	4.14 (4.12-4.16)	5.52 (5.27-5.77)	5.42 (5.31-5.52)	4.54 (4.49-4.59)	4.73 (4.66-4.80)
36-40 y	8.46 (8.42-8.49)	11.54 (11.03-12.04)	11.23 (10.99-11.48)	9.52 (9.42-9.62)	9.79 (9.64-9.95)
≥41 y	13.89 (13.83-13.95)	18.46 (17.66-19.25)	18.8 (18.42-19.17)	15.49 (15.33-15.64)	16.25 (15.99-16.50)
Overall	4.46 (4.44-4.49)	4.74 (4.50-4.98)	4.72 (4.61-4.82)	4.83 (4.77-4.89)	4.87 (4.79-4.96)
**Diabetes**					
21-25 y	0.04 (0.04-0.04)	0	0.01 (0.01-0.01)	0.06 (0.06-0.06)	0.10 (0.10-0.10)
26-30 y	0.08 (0.08-0.08)	0	0.03 (0.03-0.04)	0.13 (0.12-0.13)	0.12 (0.11-0.12)
31-35 y	0.16 (0.16-0.16)	0.02 (0.02-0.03)	0.18 (0.18-0.19)	0.46 (0.46-0.47)	0.30 (0.29-0.31)
36-40 y	0.40 (0.40-0.40)	0.64 (0.58-0.69)	1.22 (1.17-1.26)	1.31 (1.29-1.32)	0.92 (0.90-0.94)
≥41 y	0.82 (0.81-0.83)	1.31 (1.24-1.39)	2.87 (2.78-2.96)	2.30 (2.28-2.33)	1.68 (1.64-1.71)
Overall	0.23 (0.23-0.23)	0.20 (0.18-0.22)	0.43 (0.41-0.45)	0.62 (0.61-0.63)	0.43 (0.42-0.44)
**Asthma**					
21-25 y	0.41 (0.41-0.41)	0.42 (0.40-0.43)	0.44 (0.44-0.45)	0.63 (0.63-0.64)	0.55 (0.55-0.56)
26-30 y	0.61 (0.60-0.61)	0.65 (0.62-0.67)	0.66 (0.65-0.67)	1.02 (1.01-1.03)	0.81 (0.80-0.82)
31-35 y	0.96 (0.95-0.96)	1.05 (1.00-1.11)	1.02 (1.00-1.05)	1.55 (1.53-1.57)	1.22 (1.20-1.23)
36-40 y	1.22 (1.21-1.23)	1.27 (1.20-1.33)	1.31 (1.28-1.34)	1.96 (1.94-1.99)	1.53 (1.50-1.56)
≥41 y	1.23 (1.22-1.24)	1.26 (1.19-1.34)	1.28 (1.25-1.32)	1.87 (1.85-1.89)	1.46 (1.43-1.49)
Overall	0.79 (0.78-0.79)	0.75 (0.73-0.77)	0.77 (0.76-0.78)	1.24 (1.23-1.25)	1.00 (0.99-1.01)

Abbreviation: CI, confidence interval.

^a^ Adjusted for sex and rank.

^b^ Defined by the presence of appropriate *International Classification of Diseases, Ninth Revision, Clinical Modification* codes in the medical record for the preceding 24 months (12).

Compared with non-Hispanic whites, Asian/Pacific Islander and Hispanic members aged 30 or younger had a lower prevalence of hypertension. Both American Indian/Alaska Native and non-Hispanic black members had a higher prevalence of hypertension in every age category compared with non-Hispanic whites. After the age of 25, hypertension prevalence for non-Hispanic blacks was more than double that of non-Hispanic whites in every age range.

Non-Hispanic whites had a significantly lower prevalence of dyslipidemia in every age range compared with every minority racial/ethnic group.

Similar to the differences noted with hypertension, both Hispanics and non-Hispanic blacks had a significantly higher prevalence of diabetes in all age groups compared with non-Hispanic whites. After age 30, the prevalence of diabetes for non-Hispanic blacks was more than twice that of non-Hispanic whites in every age range.

The overall prevalence of asthma was significantly lower for non-Hispanic whites in every age range than for all racial/ethnic groups.

## Discussion

Compared with non-Hispanic white active-duty members in the USAF, non-Hispanic blacks are consistently and significantly more likely to be diagnosed with hypertension, dyslipidemia, diabetes, or asthma. Disparities were also noted for other racial/ethnic minorities, although these were less consistent.

The overall prevalence of hypertension in this population (5.3%) is consistent with a published prevalence of 6.7% for hypertension among 20- to 39-year-olds from the National Health and Nutrition Examination Survey (NHANES) IV (5). The NHANES IV workforce study, which was limited to employed participants in NHANES IV, found that Mexican Americans demonstrated the lowest prevalence of hypertension at 6.2%, and non-Hispanic blacks had the highest prevalence at 12.4% (6). These results are consistent with our findings that Hispanics had the lowest overall prevalence of hypertension and non-Hispanic blacks the highest. However, the fact that the prevalence of hypertension for non-Hispanic blacks was more than double that of non-Hispanic whites was a more pronounced difference and was similar to the prevalence observed among older adults (aged 65-84) in NHANES III (17).

The overall prevalence of dyslipidemia in this sample was 4.6%, which was much lower than the overall prevalence of 16.4% among employed NHANES IV participants aged 20 to 39 (6). This difference could be due to different operationalized definitions of dyslipidemia; the NHANES IV workforce study determined dyslipidemia on the basis of elevated low-density lipoprotein cholesterol results or a self-report of taking medication to lower cholesterol, rather than coded data from medical appointments. Another potential reason for the difference could be related to the entrance standards for the USAF, which exclude many risk factors that can influence the development of dyslipidemia.

The prevalence of dyslipidemia for non-Hispanic whites in our study was 4.46%, the lowest of all racial/ethnic groups. This differed from the results of the NHANES IV workforce study, which found the highest rates for non-Hispanic whites, at 18.0% (6). However, in a systematic review of cardiovascular risk factors, no minority racial/ethnic group has consistently demonstrated a higher prevalence of high cholesterol than whites (7). The NHANES IV workforce study found a 2.4% prevalence of diabetes (6). The prevalence of diabetes for active-duty USAF members was much lower, at 0.3%. This difference could be due to differing definitions of diabetes; the NHANES IV workforce study determined diabetes based on fasting blood glucose levels (6). However, a previous study found that the incidence of diabetes in the military is consistent with the incidence in a nonmilitary population (2), so the lower prevalence is likely due to pre-enlistment screening that excludes potential applicants who have already developed diabetes or have risk factors that can lead to diabetes, including an elevated body mass index. Additionally, uncontrolled diabetes is grounds for medical discharge from the USAF; some members with diabetes may have been unaccounted for by our study for this reason.

Although we found that the overall prevalence of diabetes was lower for all race/ethnicity groups than what was reported for the US population (6), the disparity for non-Hispanic blacks and Hispanics in our population is similar to what has been reported in prior studies of military (2) and nonmilitary (7) populations. However, in this sample, American Indian/Alaska Native members demonstrated a lower prevalence of diabetes compared with non-Hispanic whites. This finding is not consistent with previously published studies outside of the military (7). Pre-enlistment screening may exclude American Indian/Alaska Native members who are at higher risk for developing diabetes or these members may be more likely to develop uncontrolled diabetes and to be medically discharged; however, possible causes were beyond the scope of this study.

Prevalence of asthma in our study population was 0.9%, much lower than the overall prevalence from the Behavior Risk Factor Surveillance System of 7.7% for adults (10). This difference in overall prevalence is likely because asthma diagnosed before age 12 is a disqualification for entry into the military (11).

Despite the higher prevalence of the chronic diseases for racial or ethnic minorities compared with non-Hispanic whites, there is no evidence that that these members are less likely to participate in preventive care activities. In fact, a previous analysis from this population indicated that among active-duty members younger than 30 (more than half the sample), non-Hispanic blacks were significantly more likely to have a current preventive health assessment compared with non-Hispanic whites (*P* < .05) (18).

Outside of the military, racial/ethnic health disparities are attributed to interconnected factors, including racism (19), social and economic factors (20), and access to health care (21). Health literacy, another factor in health disparities, is also associated with poor outcomes but not necessarily with the overall use of health care (22).

The reasons for persistent racial and ethnic disparities in the prevalence of chronic diseases among a prescreened USAF population with equitable health care and living conditions are complex. However, because disparities have been identified outside of the military setting (3), pre-enlistment screening and subsequent health care and community resources likely cannot completely overcome at least 18 years of prior health neglect or culturally ingrained health habits and beliefs. In fact, several recent studies have linked childhood factors to long-term health outcomes; low socioeconomic status and experiencing a high number of adversities during childhood are associated with poor physical capabilities (23) and a high risk of developing diabetes (24).

Targeted interventions are effective at addressing disparities in the prevalence of chronic diseases (25). Just as important, however, is the need to acknowledge the effect of disparities on the overall health of men and women who enlist in the USAF and other military services. A concerted effort to understand and design culturally sensitive prevention efforts is the first step to address these disparities. Tracking existing population health metrics by race/ethnicity may also help to identify problems (and successes) and ensure that health disparities are adequately addressed.

Relying on existing clinical and administrative data has several inherent disadvantages, primarily the inability to completely account for the unique circumstances and risk factors of each person. A factor that should be considered is the overall rate of medical discharges from the military for these chronic diseases; however, these data were not available for analysis or comparison. Also, differences in personal health care–seeking behaviors may directly influence the findings because the diagnosis of these chronic diseases in this study relies on existing data from individual encounters. Some members may have met diagnostic criteria for 1 of the 4 chronic diseases included in this study but avoided screening activities.

We were, however, able to use data collected on every active-duty member in the USAF who met eligibility criteria, and to compare among all race/ethnicity, sex, and rank categories. Therefore, we were not bound by selection bias or a limited sample size. Our findings provide a reference point for future research examining health outcomes of active-duty military members by race/ethnicity.

The racial and ethnic disparities in the prevalence of the 4 chronic diseases we studied suggest the need to ensure effective preventive health care practices and community outreach efforts for racial/ethnic minorities, particularly non-Hispanic blacks.

## Acknowledgments

This study was funded by the TriService Nursing Research Program (grant no. N08-001). The Air Force Institute of Technology Civilian Institution Program and the Johns Hopkins University School of Nursing provided additional academic support.
